# N1-benzenesulfonyl-2-pyrazoline hybrids in neurological disorders: Syntheses, biological screening and computational studies

**DOI:** 10.17179/excli2017-871

**Published:** 2018-01-19

**Authors:** Avinash C. Tripathi, Savita Upadhyay, Sarvesh Paliwal, Shailendra K. Saraf

**Affiliations:** 1 Division of Pharmaceutical Chemistry, Faculty of Pharmacy, Babu Banarasi Das Northern India Institute of Technology, Lucknow-226028, U.P., India; 2 Professor and Head, Department of Pharmacy, Banasthali Vidyapith, Banasthali, Tonk-304022, Rajasthan, India

**Keywords:** 2-Pyrazolines, antidepressant and anti-anxiety, neurotoxicity, microwave synthesis, molecular docking, in silico ADME prediction

## Abstract

A novel series of 1,3,5-trisubstituted-2-pyrazolines **(5a-5t**) was prepared via Claisen Schmidt condensation, followed by heterocyclization with hydrazine hydrate, substitution of N1 hydrogen of 2-pyrazoline nucleus with 4-chlorobenzenesulfonylchloride, applying conventional and green chemistry approaches. Among the two, microwave assisted organic synthesis (MAOS) emerged as a better synthetic tool in terms of faster reaction rate and high yield. Various physicochemical and spectral studies were conducted to characterize the synthesized derivatives including- IR, Mass, ^1^H-NMR, ^13^C-NMR and elemental analysis. During pharmacological evaluation, compound **5b **showed excellent anti-anxiety activity and compound **5k** exhibited the best antidepressant effect at the tested doses, 50 and 100 mg/kg b.w., being comparable to diazepam and imipramine, respectively. The docking experiments confirmed the probable mechanism of neuropharmacological action, showing excellent affinity towards MAO-A target protein, which was also evidenced from some of the key interactions with binding site residues Ala68, Tyr69 and Phe352. Furthermore, complimentary *in silico* pharmacokinetic recital without any potential risk of neurotoxicity (as evaluated by rotarod and actophotometer tests), or carcinogenicity, mutagenicity, reproductive toxicity, acute toxicity and irritancy (as predicted by LAZAR and OSIRIS programs) signified their probable use in depression and anxiety disorders.

## Introduction

Monoamine oxidase (MAO) regulates monoaminergic homeostasis and neurotransmission in the nervous system. Low level of certain neurotransmitters (NTs) in the brain, like dopamine (DA), norepinephrine (NE), serotonin (5-HT), and gamma amino butyric acid (GABA), is the main cause of depressive mental disorders. These NTs are released during neurotransmission and are degraded by the MAOs enzymes. Monoamine oxidase inhibitors (MAOIs) block the action of MAOs, and thus increase the concentration of NTs in the brain (Mitoma and Ito, 1992[26]; Meyer et al., 2006[24]). The antidepressant effect of a hydrazine based anti-tubercular drug, iproniazid (a structural analog of isoniazid) started the journey of development of MAOIs. This discovery showed the way to design and produce some more effective analogs such as phenelzine (Zeller and Barsky, 1952[46]). MAOIs belong to the first generation antidepressants used for decades to treat the patients suffering from high level of anxiety, atypical depression (Pletscher, 1991[32]), anergic bipolar depression and treatment resistant depression (Thase, 2012[39]), specific phobias, post-traumatic stress disorder and migraine headaches resistant to other therapies (Gareri et al., 2000[11]). Two major therapeutic categories of MAOI: the MAO-A inhibitors in certain mental disorders such as depression and anxiety (Amrein et al., 1999[2]) and MAO-B inhibitors proved their remedial value in neurodegenerative diseases (Youdim et al., 2006[45]; Foley et al., 2000[10]) including Parkinson's (Cesura and Pletscher, 1992[5]) and Alzheimer's (Volz and Gleiter, 1998[43]). The initial hydrazine class of MAO inhibitors was associated with some severe adverse effects such as liver toxicity and cheese reaction (Brown et al., 1989[4]). These side-effects were correlated to nonselective and irreversible MAO inhibition. These findings prompted research to develop selective and reversible type of MAO (A and B) inhibitors. Pyrazoline derivatives have attracted substantial attention for years, chiefly 1,3,5-trisubstituted-2-pyrazoline pharmacophore is allied with encouraging neurological activities such as tranquilizer, anticonvulsant and antidepressant (Kaplancikli et al., 2010[17]; Ozdemir et al., 2008[28]; Palaska et al., 2001[29]; Rajendra Prasad et al., 2005[35]; Ruhoglu et al., 2005[36]), psychoanaleptic, MAO inhibitory and other biological activities (Chimenti et al., 2004[6]; 2010[7]; Gokhan et al., 2003[13]; Gokhan-Kelekci et al., 2009[14]; Jagrat et al., 2011[15]; Jayaprakash et al., 2008[16]; Karuppasamy et al., 2010[18]; Maccioni et al., 2010[22]; Manna et al., 2002[23]; Mishra and Sasmal, 2011[25]; Sahoo et al., 2010[37]; Agrawal et al., 2012[1]; Gautam et al., 2010[12]). Considering these facts, some 1,3,5-trisubstituted-2-pyrazolines were synthesized by integrating some novel aromatic and hetero-aromatic substitutions at 3^rd^ and 5^th^ positions, respectively. Microwave facilitated synthetic technique was also utilized to prepare the proposed compounds and the results were compared to that of the conventional heating methods (Lidstrom et al., 2001[20]). This work is in continuation to the previous communications by our group (Tripathi et al., 2016[40], Upadhyay et al., 2017[41]; Bhandari et al., 2013[3]) with some novel derivatives having promising potential in the field.

## Materials and Methods

S. D. Fine Chemicals and Sigma Aldrich, Mumbai, India supplied the chemicals and reagents for synthesis and the pre-coated TLC sheets were obtained from Merck Chemicals, India and were used as such. Microwave assisted organic synthesis (MAOS) was performed on Raga's Scientific Microwave System (Ragatech, Pune, Maharashtra, India). 

### Chemistry

**Figure 1:**
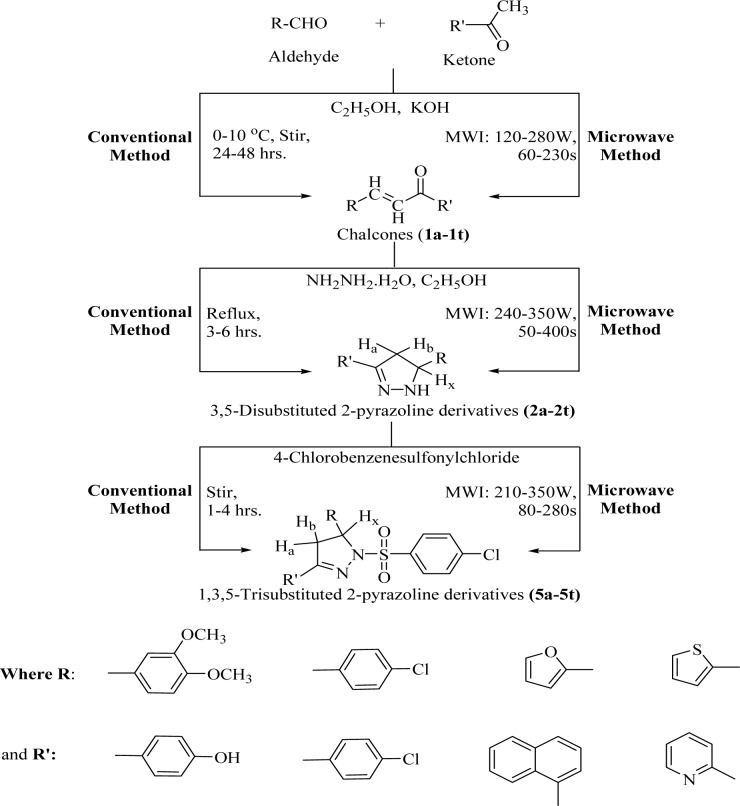
Synthesis of 1,3,5-Trisubstituted-2-pyrazoline derivatives

The reaction of suitably substituted aromatic/heteroaromatic ketones with different aldehydes, in alkaline medium, yielded substituted chalcones (**1a-1t**) via Claisen-Schmidt condensation. Then, the formed chalcones were reacted with hydrazine hydrate (in excess) to give 2-pyrazoline derivatives (**2a-2t**). Finally, the N1 hydrogen of the 2-pyrazoline nucleus was replaced with 4-chlorobenzenesulfonylchloride (**5a-5t**), as given in Figure 1(Fig. 1). The reaction progress was examined by thin layer chromatography (TLC), using pre-coated silica gel G plates as stationary phase and iodine vapors/UV light as the visualizing agents.

### Synthesis of chalcone derivatives (1a-1t)

The proposed chalcone derivatives of the first step were synthesized by reacting suitably substituted aldehydes and ketones, in equimolar ratio, via conventional and microwave assisted organic synthesis (MAOS) procedures (Tripathi et al., 2016[40]; Upadhyay et al., 2017[41]; Agrawal et al., 2012[1]; Gautam et al., 2010[12]). 

### Synthesis of 3,5-disubstituted-2-pyrazoline derivatives(2a-2t)

The 3,5-disubstituted-2-pyrazoline derivatives of the second step were synthesized by reacting different chalcone derivatives of the first step with hydrazine hydrate (excess), using conventional and MAOS procedures (Tripathi et al., 2016[40]; Upadhyay et al., 2017[41]).

### Synthesis of 1,3,5-trisubstituted-2-pyrazoline derivatives (5a-5t)

*Conventional synthesis:* Appropriately substituted 2-pyrazoline derivatives (0.001M) of the second step reacted with 4-chlorobenzenesulfonylchloride (0.002M) by stirring, using THF (10 mL) as the solvent. Continued the stirring for 1-4 hrs, poured the reaction mixture in a petri plate and evaporated the solvent up to dryness. Re-precipitated the crude product using acetonitrile/methanol and re-crystallized from acetonitrile/methanol to obtain the pure product (Upadhyay et al., 2017[41]). 

*MAOS:* Appropriately substituted 3,5-disubstituted-2-pyrazolines (0.001M) were reacted with 4-chlorobenzenesulfonylchloride (0.002M) under microwave irradiation (MWI: 210-350W; 80-280s), taking THF (10 mL) as the solvent. The reaction mixture was poured in a petri plate and evaporated the solvent up to dryness. The crude product was re-precipitated using acetonitrile/methanol and recrystallized from acetonitrile/methanol to get the purified derivative (Upadhyay et al., 2017[41]).

### Characterization of the synthesized 1,3,5-trisubstituted-2-pyrazoline derivatives

First and second step intermediate compounds were characterized by TLC, melting point (determined by open capillary method and are uncorrected) and mass spectrometric techniques. However, comprehensive physicochemical (Table 1(Tab. 1)) and spectral characterization was undertaken for the final derivatives and the values were found to be in agreement. 

#### 4-[1-(4-Chloro-benzenesulfonyl)-5-(3,4-dimethoxy-phenyl)-4,5-dihydro-1H-pyrazol-3-yl]-phenol (5a)

IR spectra were recorded on Bruker FT-IR, ALPHA-T (Eco-ATR) spectrophotometers, (Bruker Corporation., USA) and the values are expressed in cm^-1^. 3220 (N-H stretch), 2865 (C-H Aromatic), 1650 (C=N stretch), 1515 (C-H deform), 1159, 1350 (sym., asym S(=O)_2_ stretch). ^1^H-NMR and ^13^C-NMR spectra were recorded on Bruker Avance-400, FTNMR spectrometer (Bruker, Tech. Pvt. Ltd., USA) at 400MHz and the chemical shifts are reported in parts per million (δ value), taking TMS (δ 0 ppm for ^1^H NMR) as the internal standard: 2.03-2.09 (dd, *J*_ab_: 16.77 Hz, *J*_ax_: 3.58 Hz, 1H, H_a_), 2.73-2.78 (dd, *J*_ab_: 3.85 Hz, *J*_bx_: 17.11 Hz, 1H, H_b_), 3.81-3.93 (dd, *J*_ax_: 3.50 Hz, *J*_bx_: 17.05 Hz, 1H, H_x_), 3.97-4.10 (m, 6H, methyl), 5.23-5.25 (s, 1H, Ar-OH), 6.56-7.90 (m, 11H, Ar). ^13^C NMR (DMSO, ppm): 37.9 (CH_2_ pyrazoline), 43.0 (CH pyrazoline), 113.8-146.3 (12CH benzene), 147.5-159.2 (7C benzene), 160.1 (C pyrazoline). Mass spectra were recorded on Waters UPLC-TQD Mass Spectrometer instrument (Waters Corporation, USA) using LC-ESI or APCI-MS Technique; MS (*m/z*): 473 (M^+^, 100 %). Elemental analysis was performed on Perkin Elmer-2400, Series-II Analyzer (Waltham, Massachusetts, USA). Anal. Calcd. for C_23_H_21_ClN_2_O_5_S: C, 58.41; H, 4.48; N, 5.92. Found: C, 58.39; H, 4.50; N, 5.89. 

#### 4-[1-(4-Chloro-benzenesulfonyl)-5-(4-chloro-phenyl)-4,5-dihydro-1H-pyrazol-3-yl]-phenol (5b)

IR (cm^-1^): 3359 (N-H stretch), 3142 (C-H Aromatic), 1606 (C=N stretch), 1516 (C-H deform), 1165, 1351 (sym., asym S(=O)_2_ stretch). ^1^H-NMR ( δ ppm, DMSO): 1.98-1.99 (dd, *J*_ab_: 17.12 Hz, *J*_ax_: 3.41 Hz, 1H, H_a_), 3.40-3.48 (dd, *J*_ab_: 4.01 Hz, *J*_bx_: 16.23 Hz, 1H, H_b_), 3.70-3.80 (dd, *J*_ax_: 3.28 Hz, *J*_bx_: 17.46 Hz, 1H, H_x_), 5.39-5.45 (s, 1H, Ar-OH), 6.88-7.89 (m, 12H, Ar). ^13^C NMR (DMSO, ppm): 38.6 (CH_2_ pyrazoline), 43.2 (CH pyrazoline), 115.6-140.3 (12CH benzene), 158.3-160.1 (3C benzene), 161.2 (C pyrazoline). MS (*m/z*): 447 (M^+^, 100 %). Anal. Calcd. for C_21_H_16_Cl_2_N_2_O_3_S: C, 56.38; H, 3.61; N, 6.26. Found: C, 57.42; H, 3.59; N, 6.24.

#### 4-[1-(4-Chloro-benzenesulfonyl)-5-furan-2-yl-4,5-dihydro-1H-pyrazol-3-yl]-phenol (5c)

IR (cm^-1^): 3472 (N-H stretch), 3239 (C-H Aromatic), 1602 (C=N stretch), 1434 (C-H deform), 1159, 1348 (sym., asym S(=O)_2_ stretch). ^1^H NMR (δ ppm, DMSO): 2.14-2.16 (dd, *J*_ab_: 17.00 Hz, *J*_ax_: 3.18 Hz, 1H, H_a_), 3.27-3.36 (dd, *J*_ab_: 3.88 Hz, *J*_bx_: 16.69 Hz, 1H, H_b_), 3.42-3.84 (dd, *J*_ax_: 3.05 Hz, *J*_bx_: 16.96 Hz, 1H, H_x_), 5.20 (s, 1H, Ar-OH), 6.04-7.66 (m, 11H, Ar). ^13^C NMR (DMSO, ppm): 40.1 (CH_2_ pyrazoline), 44.5 (CH pyrazoline), 104.2-142.9 (11CH Ar), 135.3-158.6 (4C benzene), 159.0 (C furan), 160.7 (C pyrazoline). MS (*m/z*): 229 (M^+^, 100 %). Anal. Calcd. for C_19_H_15_ClN_2_O_4_S: C, 56.65; H, 3.75; N, 6.95. Found: C, 56.66; H, 3.77; N, 6.93. 

#### 4-[1-(4-Chloro-benzenesulfonyl)-5-thiophen-2-yl-4,5-dihydro-1H-pyrazol-3-yl]-phenol (5d)

IR (cm^-1^): 3270 (N-H stretch), 3010 (C-H Aromatic), 1615 (C=N stretch), 1463 (C-H deform), 1189, 1382 (sym., asym S(=O)_2_ stretch). ^1^H NMR (δ ppm, DMSO): 1.99-2.00 (dd, *J*_ab_: 15.72 Hz, *J*_ax_: 3.86 Hz, 1H, H_a_), 3.40-3.48 (dd, *J*_ab_: 3.94 Hz, *J*_bx_: 16.70 Hz, 1H, H_b_), 3.71-3.85 (dd, *J*_ax_: 3.28 Hz, *J*_bx_: 17.46 Hz, 1H, H_x_), 5.45 (s, 1H, Ar-OH), 6.75-7.80 (m, 11H, Ar). ^13^C NMR (DMSO, ppm): 39.4 (CH_2_ pyrazoline), 44.8 (CH pyrazoline), 113.8-138.6 (11CH Ar), 120.1-151.7 (4C benzene), 161.3 (C thiophene), 162.4 (C pyrazoline). MS (*m/z*): 419 (M^+^, 95 %). Anal. Calcd. for C_19_H_15_ClN_2_O_3_S_2_: C, 54.47; H, 3.61; N, 6.69. Found: C, 54.43; H, 3.62; N, 6.67.

#### 1-(4-Chloro-benzenesulfonyl)-3,5-bis-(4-chloro-phenyl)-4,5-dihydro-1H-pyrazole (5f)

IR (cm^-1^): 3315 (N-H stretch), 3100 (C-H Aromatic), 1616 (C=N stretch), 1465 (C-H deform), sym., asym S(=O)_2_ stretch (1164, 1389). ^1^H NMR (δ ppm, DMSO): 1.91-1.96 (dd, *J*_ab_: 17.56 Hz, *J*_ax_: 3.50 Hz, 1H, H_a_), 3.29-3.48 (dd, *J*_ab_: 4.01 Hz, *J*_bx_: 17.23 Hz, 1H, H_b_), 3.66-3.84 (dd, *J*_ax_: 3.75 Hz, *J*_bx_: 16.54 Hz, 1H, H_x_), 7.00-7.77 (m, 12H, Ar). ^13^C NMR (DMSO, ppm): 39.0 (CH_2_ pyrazoline), 42.5 (CH pyrazoline), 125.8-131.2 (12CH benzene), 136.1-141.9 (4C benzene), 161.2 (C pyrazoline). MS (*m/z*): 463 (M^+^, 55 %). Anal. Calcd. for C_21_H_15_Cl_3_N_2_O_2_S: C, 54.15; H, 3.25; N, 6.01. Found: C, 54.11; H, 3.22; N, 6.00. 

#### 1-(4-Chloro-benzenesulfonyl)-3-(4-chloro-phenyl)-5-furan-2-yl-4,5-dihydro-1H-pyrazole (5g)

IR (cm^-1^): 3330 (N-H stretch), 3092 (C-H Aromatic), 1685 (C=N stretch), 1539 (C-H deform), 1168, 1355 (sym., asym S(=O)_2_ stretch). ^1^H NMR (δ ppm, DMSO): 2.08-2.13 (dd, *J*_ab_: 15.31 Hz, *J*_ax_: 2.98 Hz, 1H, H_a_), 3.39-3.44 (dd, *J*_ab_: 4.16 Hz, *J*_bx_: 15.57 Hz, 1H, H_b_), 3.61-3.79 (dd, *J*_ax_: 3.58 Hz, *J*_bx_: 16.95 Hz, 1H, H_x_), 6.53-7.60 (m, 11H, Ar). ^13^C NMR (DMSO, ppm): 39.9 (CH_2_ pyrazoline), 42.5 (CH pyrazoline), 107.7-128.5 (11CH Ar), 130.2-136.8 (3C benzene), 159.0 (C furan), 161.7 (C pyrazoline). MS (*m/z*): 421 (M^+^, 65 %). Anal. Calcd. for C_19_H_14_Cl_2_N_2_O_3_S: C, 54.17; H, 3.35; N, 6.65. Found: C, 54.20; H, 3.39; N, 6.61. 

#### 1-(4-Chloro-benzenesulfonyl)-3-(4-chloro-phenyl)-5-thiophen-2-yl-4,5-dihydro-1H-pyrazole (5h)

IR (cm^-1^): 3345 (N-H stretch), 3056 (C-H Aromatic), 1621 (C=N stretch), 1471 (C-H deform), 1165, 1393 (sym., asym S(=O)_2_ stretch). ^1^H NMR (δ ppm, DMSO): 2.10-2.19 (dd, *J*_ab_: 16.88 Hz, *J*_ax_: 3.29 Hz, 1H, H_a_), 4.15-4.22 (dd, *J*_ab_: 4.01 Hz, *J*_bx_: 16.78 Hz, 1H, H_b_), 4.20-4.23 (dd, *J*_ax_: 3.63 Hz, *J*_bx_: 16.91 Hz, 1H, H_x_), 6.65-7.79 (m, 11H, Ar). ^13^C NMR (DMSO, ppm): 39.7 (CH_2_ pyrazoline), 41.3 (CH pyrazoline), 121.4-136.5 (11CH Ar), 128.6-134.1 (3C benzene), 139.4 (C thiophene), 155.8 (C pyrazoline). MS (*m/z*): 437 (M^+^, 40 %). Anal. Calcd. for C_19_H_14_Cl_2_N_2_O_2_S_2_: C, 52.18; H, 3.23; N, 6.41. Found: C, 52.17; H, 3.20; N, 6.47. 

#### 1-(4-Chloro-benzenesulfonyl)-5-(3,4-dimethoxy-phenyl)-3-naphthalen-1-yl-4,5-dihydro-1H-pyrazole (5i)

IR (cm^-1^): 3345 (N-H stretch), 3056 (C-H Aromatic), 1577 (C=N stretch), 1462 (C-H deform), 116570, 1362 (sym., asym S(=O)_2_ stretch). ^1^H NMR (δ ppm, DMSO): 1.97-1.98 (dd, *J*_ab_: 17.22 Hz, *J*_ax_: 3.52 Hz, 1H, H_a_), 3.39-3.47 (dd, *J*_ab_: 3.87 Hz, *J*_bx_: 16.73 Hz, 1H, H_b_), 3.54-3.69 (dd, *J*_ax_: 3.18 Hz, *J*_bx_: 17.26 Hz, 1H, H_x_), 3.80-3.94 (m, 6H, methyl), 6.78-7.64 (m, 14H, Ar). ^13^C NMR (DMSO, ppm): 39.7 (CH_2_ pyrazoline), 42.5 (CH pyrazoline), 61.7 (2CH_3_), 113.7-132.5 (14CH Ar), 130.8-137.2 (7C Ar), 159.3 (C pyrazoline). MS (*m/z*): 507 (M^+^, 50 %). Anal. Calcd. for C_27_H_23_ClN_2_O_4_S: C, 63.96; H, 4.57; N, 5.53. Found: C, 64.07; H, 4.61; N, 5.46. 

#### 1-(4-Chloro-benzenesulfonyl)-5-(4-chloro-phenyl)-3-naphthalen-1-yl-4,5-dihydro-1H-pyrazole (5j)

IR (cm^-1^): 3266 (N-H stretch), 3011 (C-H Aromatic), 1570 (C=N stretch), 1495 (C-H deform), 1166, 1362 (sym., asym S(=O)_2_ stretch). ^1^H NMR (δ ppm, DMSO): 2.15-2.19 (dd, *J*_ab_: 16.68 Hz, *J*_ax_: 3.27 Hz, 1H, H_a_), 3.83-3.88 (dd, *J*_ab_: 3.95 Hz, *J*_bx_: 17.10 Hz, 1H, H_b_), 3.50-3.67 (dd, *J*_ax_: 4.12 Hz, *J*_bx_: 16.97 Hz, 1H, H_x_), 6.90-7.73 (m, 14H, Ar). ^13^C NMR (DMSO, ppm): 39.2 (CH_2_ pyrazoline), 46.4 (CH pyrazoline), 124.2-129.0 (14CH Ar), 128.5-136.1 (7C benzene), 158.7 (C pyrazoline). MS (*m/z*): 481 (M^+^, 50 %). Anal. Calcd. for C_25_H_18_Cl_2_N_2_O_2_S: C, 62.37; H, 3.77; N, 5.82. Found: C, 62.49; H, 3.80; N, 5.78. 

#### 1-(4-Chloro-benzenesulfonyl)-5-furan-2-yl-3-naphthalen-1-yl-4,5-dihydro-1H-pyrazole (5k)

IR (cm^-1^): 3290 (N-H stretch), 3050 (C-H Aromatic), 1635 (C=N stretch), 1469 (C-H deform), 1166, 1355 (sym., asym S(=O)_2_ stretch). ^1^H NMR (δ ppm, DMSO): 2.48-2.51 (dd, *J*_ab_: 17.12 Hz, *J*_ax_: 3.41 Hz, 1H, H_a_), 3.04-3.89 (dd, *J*_ab_: 4.01 Hz, *J*_bx_: 16.23 Hz, 1H, H_b_), 4.88-5.21 (dd, *J*_ax_: 3.28 Hz, *J*_bx_: 17.46 Hz, 1H, H_x_), 6.80-6.93 (d, 2H, Ar), 7.43-7.54 (m, 4H, Ar), 7.66-7.85 (m, 7H, Ar). ^13^C NMR (DMSO, ppm): 50.7 (CH_2_ pyrazoline), 77.2 (CH pyrazoline), 124.4-126.9 (2CH furan), 129.0-131.3 (7CH naphthalene), 133.7-138.2 (4CH benzene), 159.4 (C pyrazoline). MS (*m/z*): 437 (M^+^, 50 %), 438 (30 %). Anal. Calcd. for C_23_H_17_ClN_2_O_3_S: C, 63.23; H, 3.92; N, 6.41. Found: C, 63.26; H, 3.97; N, 6.40. 

#### 1-(4-Chloro-benzenesulfonyl)-3-naphthalen-1-yl-5-thiophen-2-yl-4,5-dihydro-1H-pyrazole (5l)

IR (cm^-1^): 3292 (N-H stretch), 3047 (C-H Aromatic), 1581 (C=N stretch), 1509 (C-H deform), 1177, 1322 (sym., asym S(=O)_2_ stretch). ^1^H NMR (δ ppm, DMSO): 2.08-2.09 (dd, *J*_ab_: 17.48 Hz, *J*_ax_: 2.99 Hz, 1H, H_a_), 3.10-3.18 (dd, *J*_ab_: 3.74 Hz, *J*_bx_: 17.04 Hz, 1H, H_b_), 3.83-3.86 (dd, *J*_ax_: 3.26 Hz, *J*_bx_: 16.36 Hz, 1H, H_x_), 6.60-7.92 (m, 14H, Ar). ^13^C NMR (DMSO, ppm): 40.6 (CH_2_ pyrazoline), 46.1 (CH pyrazoline), 118.5-129.2 (14CH Ar), 137.8-148.1 (6C Ar), 161.5 (C pyrazoline). MS (*m/z*): 463 (M^+^, 50 %). Anal. Calcd. for C_23_H_17_ClN_2_O_2_S_2_: C, 60.98; H, 3.78; N, 6.18. Found: C, 61.06; H, 3.76; N, 6.21. 

#### 2-[1-(4-Chloro-benzenesulfonyl)-5-(3,4-dimethoxy-phenyl)-4,5-dihydro-1H-pyrazol-3-yl]-pyridine (5q)

IR (cm^-1^): 3321 (N-H stretch), 3084 (C-H Aromatic), 1624 (C=N stretch), 1466 (C-H deform), 1170, 1337 (sym., asym S(=O)_2_ stretch). ^1^H NMR (δ ppm, DMSO): 1.89-1.91 (dd, *J*_ab_: 15.62 Hz, *J*_ax_: 3.17 Hz, 1H, H_a_), 3.33-3.39 (dd, *J*_ab_: 3.74 Hz, *J*_bx_: 17.11 Hz, 1H, H_b_), 3.65-3.80 (dd, *J*_ax_: 3.54 Hz, *J*_bx_: 16.91 Hz, 1H, H_x_), 3.73-3.94 (m, 6H, methyl), 6.97-8.21 (m, 11H, Ar). ^13^C NMR (DMSO, ppm): 40.2 (CH_2_ pyrazoline), 45.7 (CH pyrazoline), 49.5 (6C, CH_3_), 114.9-135.8 (11CH Ar), 143.4-152.8 (6C benzene), 161.9 (C pyrazoline). MS (*m/z*): 458 (M^+^, 30 %). Anal. Calcd. for C_22_H_20_ClN_3_O_4_S: C, 57.70; H, 4.40; N, 9.18. Found: C, 57.64; H, 4.45; N, 9.14. 

#### 2-[1-(4-Chloro-benzenesulfonyl)-5-(4-chloro-phenyl)-4,5-dihydro-1H-pyrazol-3-yl]-pyridine (5r)

IR (cm^-1^): 3421 (N-H stretch), 3091 (C-H Aromatic), 1616 (C=N stretch), 1471 (C-H deform), 1170,2 1383 (sym., asym S(=O)_2_ stretch). ^1^H NMR (δ ppm, DMSO): 1.91-1.94 (dd, *J*_ab_: 17.36 Hz, *J*_ax_: 3.21 Hz, 1H, H_a_), 3.64-3.48 (dd, *J*_ab_: 4.12 Hz, *J*_bx_: 17.44 Hz, 1H, H_b_), 3.69-3.85 (dd, *J*_ax_: 3.28 Hz, *J*_bx_: 16.55 Hz, 1H, H_x_), 7.29-8.69 (m, 12H, Ar). ^13^C NMR (DMSO, ppm): 40.2 (CH_2_ pyrazoline), 44.5 (CH pyrazoline), 124.6-149.2 (12CH Ar), 137.1-139.4 (2C benzene), 154.2 (C pyridine), 160.7 (C pyrazoline). MS (*m/z*): 432 (M^+^, 55%). Anal. Calcd. for C_20_H_15_Cl_2_N_3_O_2_S: C, 55.56; H, 3.50; N, 9.72. Found: C, 55.60; H, 3.51; N, 9.68. 

#### 2-[1-(4-Chloro-benzenesulfonyl)-5-thiophen-2-yl-4,5-dihydro-1H-pyrazol-3-yl]-pyridine (5t)

IR (cm^-1^): 3387 (N-H stretch), 3083 (C-H Aromatic), 1612 (C=N stretch), 1468 (C-H deform), 1167, 1389 (sym., asym S(=O)_2_ stretch). ^1^H NMR (δ ppm, DMSO): 1.90-1.93 (dd, *J*_ab_: 17.23 Hz, *J*_ax_: 3.32 Hz, 1H, H_a_), 3.46-3.50 (dd, *J*_ab_: 3.87 Hz, *J*_bx_: 16.65 Hz, 1H, H_b_), 3.82-3.89 (dd, *J*_ax_: 3.14 Hz, *J*_bx_: 16.91 Hz, 1H, H_x_), 6.53-8.97 (m, 11H, Ar). ^13^C NMR (DMSO, ppm): 38.6 (CH_2_ pyrazoline), 43.2 (C pyrazoline), 115.6-140.3 (12CH benzene), 144.9-157.1 (4C Ar), 161.3 (C pyrazoline). MS (*m/z*): 403 (M^+^, 25 %). Anal. Calcd. for C_18_H_14_ClN_3_O_2_S_2_: C, 53.53; H, 3.49; N, 10.40. Found: C, 53.49; H, 3.53; N, 10.37. 

### Biological evaluation

#### Study animals

The study protocols were authenticated by the Institutional Animal Ethics Committee (IAEC) with the protocol number BBDNIIT/IAEC/009/2014. 

Animal house, Faculty of Pharmacy, BBDNIIT, Lucknow, U.P., India provided animals (male albino mice) for the *in-vivo *study which were treated humanely and maintained under standard conditions of temperature (26 ± 2 ºC), percent humidity (55 ± 5), light and dark cycles and free access to food and water. 

#### Study design

The studies were performed as per reported procedures (Tripathi et al., 2016[40]; Upadhyay et al., 2017[41]). The detailed description of the groups and respective doses are given in Table 2(Tab. 2).

#### Antidepressant activity

The antidepressant potential of the synthesized derivatives (**5a-5t**) was evaluated using behavioral *in vivo *tests in mice model, such as forced swim test (FST) and tail suspension test, (TST) as given by Porsolt (1981[33]) and Willner and Mitchell (2002[44]).

##### Porsolt's behavioral despair or FST 

In FST, the state of despair can be minimized by different therapeutically effective antidepressants (Porsolt et al., 1977[34]; Vogel, 2002[42]). The duration of immobility in animals, produced by the test compounds, was measured by known procedures (Tripathi et al., 2016[40]; Upadhyay et al., 2017[41]).

##### Tail suspension test (TST) 

TST is another well known method (Steru et al., 1985[38]; Vogel, 2002[42]), employed to evaluate the antidepressant potential of the test compounds. In this test, the duration of immobility was measured by known procedures (Tripathi et al., 2016[40]; Upadhyay et al., 2017[41]).

### Anti-anxiety activity

The maze model was used to evaluate anti-anxiety potential of the test compounds, which selectively identified the anxiolytic (open arm exploration time increased and time spent in closed arms decreased) and anxiogenic drugs (Vogel, 2002[42]).

#### Elevated plus-maze test

The elevated plus-maze was used to determine anxiety-related behavior, measured by the extent to which the mouse avoids visiting the open arm of the maze (Lister, 1987[21]; Pellow et al., 1985[31]). After one hour of oral administration of the test compound, observations (i.e. time spent in closed arm and total number of the arm entries) were recorded during the 6 minute test duration by known procedures (Tripathi et al., 2016[40]; Upadhyay et al., 2017[41]).

### Neurotoxicity study 

This study was used to evaluate the effect of the compounds on the CNS, employing various behavioral tests, such as rotarod, open-field/actophotometer, turning on flat surface and inclined plane tests in mice model (Vogel, 2002[42]; Parasuraman, 2011[30]).

### Neuromuscular coordination study

The effect of the compounds on motor coordination, where relaxation of skeletal muscle is produced, was examined, as per method given by Dunham and Miya in 1957[9], with slight modification (Vogel et al., 2002[42]). 

#### Locomotor activity (actophotometer test)

Locomotor activities of the animals were used as an index of their mental alertness, which is affected (increased or decreased) by majority of the CNS acting drugs (Dhingra and Goyal, 2008[8]; Tripathi et al., 2016[40]; Upadhyay et al., 2017[41]).

### Acute toxicity study

The consequences (changes in skin and fur, behavior patterns, convulsions, tremors and death) of a single dose on a particular animal species was determined by acute toxicity testing. In this study, acute oral toxicity (LD_50_) of two most active final derivatives (**5b** and **5k**) was performed as per guidelines laid by Organization for Economic Cooperation and Development (OECD) guideline No 423 “Acute Oral Toxicity - Acute Toxic Class Method” using known procedures (OECD, 2001[27]).

### Computational studies

Molecular docking simulations were used to predict binding affinity and binding orientations of the synthesized compounds to the MAO-A protein (PDB ID: 2Z5X), with the help of GLIDE program (Schrödinger, LLC, 2014), using known procedures (Tripathi et al., 2016[40]; Upadhyay et al., 2017[41]). QikProp module of Schrödinger software program was successfully employed for *in silico* prediction of ADME properties of the synthesized derivatives. Furthermore, for *in silico *toxicity prediction of the synthesized compounds, two freely accessible computer programs (LAZAR and OSIRIS Property explorer) were employed. Among them, LAZAR provided a generic tool for predicting complex toxicological end points (like long-term toxicity, reproductive toxicity and carcinogenicity). OSIRIS program calculated various drug-related properties of chemical structures and risks of untoward effects, which are considered as an indication of drug-conform behavior (Klebe, 2000[19]).

### Statistical analysis

Statistical analysis was carried out using Graph Pad Prism 5.0 (Graph Pad Software, San Diego, CA). Experimental results are expressed as mean±SD, analyzed by one-way ANOVA followed by Dunnett's test for the possible significance (P<0.05) between various groups.

## Results and Discussion

A total of fourteen 1,3,5-trisubstituted 2-pyrazoline derivatives **(5a-5t) **were prepared through Claisen-Schmidt condensation, followed by heterocyclization and substitution with 4-chlorobenzenesulfonylchloride using both, conventional and MAOS methods. The microwave assisted procedures were developed for the first time to synthesize the final 1,3,5-trisubstituted-2-pyrazoline derivatives. The research outcomes suggested that almost all the derivatives synthesized by this technique were obtained at a faster rate and in a better synthetic yield in comparison to conventional procedures. The prepared derivatives were analyzed by various physicochemical (Table 1(Tab. 1)) and spectral techniques and results comply with the proposal. The IR spectra reflect some characteristic absorption bands in the corresponding regions to C=N str (1509-1612 cm^-1^), N-H str (3449-3107 cm^-1^) and C-H deform (1431-1354 cm^-1^). In the third step derivatives, a characteristic peak of sulfonyl group was also observed in the IR spectra. ^1^H-NMR spectra showed that two dissimilar methylene protons (H_a_/H_b_) were visible at δ 2.92-3.38 ppm, 3.70-3.93 which coupled themselves and with the methine proton (H_x_) at δ 6.67-7.03 in vicinity. The other values were in congruence with the corresponding aliphatic and aromatic protons.

**Figure 2:**
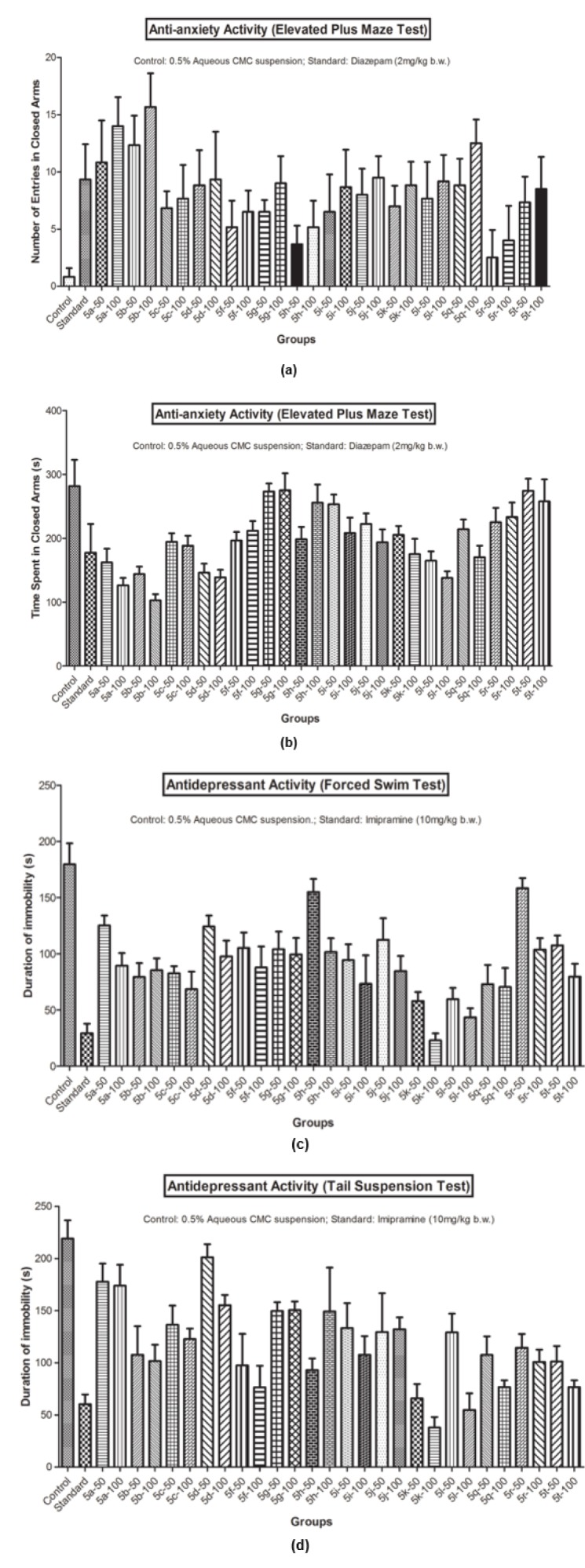
(a) Anti-anxiety activity (Number of entries in closed arms in Elevated Plus Maze Test); (b) Anti-anxiety activity (Time spent in closed arms in Elevated Plus Maze Test); (c) Antidepressant activity (Forced Swim Test); (d) Antidepressant activity (Tail Suspension Test)

The pharmacological results (Table 2(Tab. 2) and Figure 2(Fig. 2)) demonstrated that number of entries in closed and open arms, in the elevated plus maze test, increased significantly with a lesser closed arm exploration time (Figure 2a and 2b(Fig. 2)) for Compound **5b** and Compound **5k** showed the best antidepressant action (Figure 2c and 2d(Fig. 2)) in FST and TST models at the evaluated doses. The pharmacological actions of the prepared analogs were observed in a dose dependent manner, as the magnitude of the effects increased at higher tested doses (100 mg/kg b.w.). The structure activity relationship studies established that C-3 naphthalen-1-yl substitution and C-5 furan-2-yl substitution at 2-pyrazoline nucleus were crucial for the antidepressant activity (**5k**). However, a polar substitution of 4-hydroxyphenyl group at 3^rd^ position (**5b**) was essential in eliciting anxiolytic activity.

**Figure 3:**
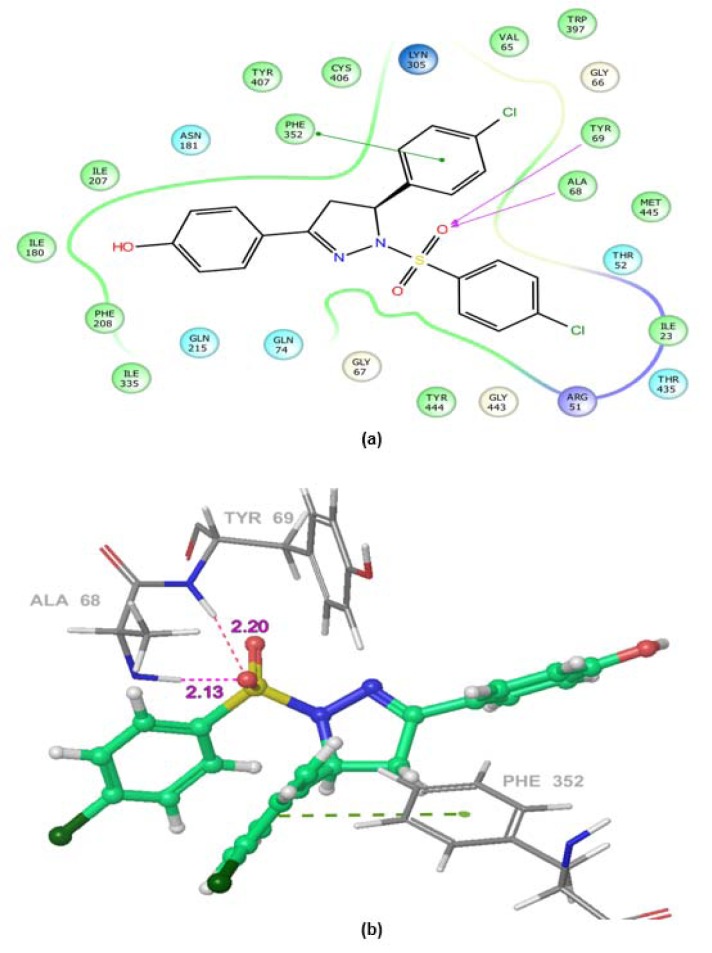
Ligand receptor interaction diagram of compound 5b at the binding site of MAO-A protein (PDB ID: 2Z5X) showing best anti-anxiety activity. (a) 2D Ligand receptor interaction diagram. (b) 3D Ligand receptor interaction diagram.

**Figure 4:**
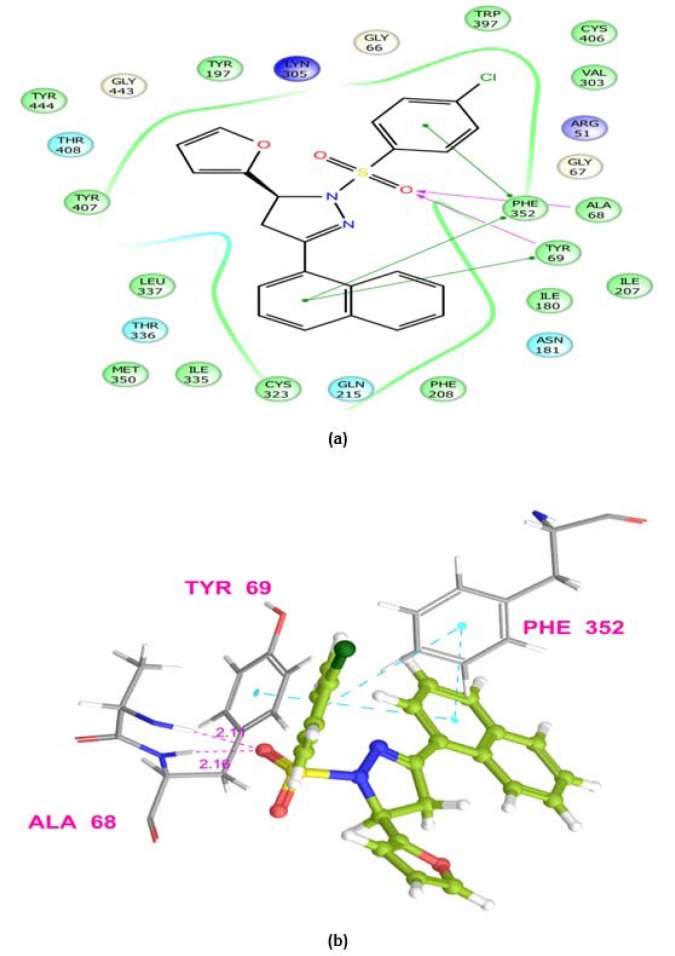
Ligand receptor interaction diagram of compound 5k at the binding site of MAO-A protein (PDB ID: 2Z5X) showing best antidepressant activity. (a) 2D Ligand receptor interaction diagram. (b) 3D Ligand receptor interaction diagram.

MAOs are considered as an important biological target to develop drugs against certain mental disorders, such as depression and anxiety. It has been also established that the 2-pyrazoline derivatives possess excellent affinity towards MAO isoforms, as reported in previous literatures. Docking studies allowed to gain some structural inputs towards binding characteristics of the prepared derivatives with the MAO-A target protein. The most stable conformation of ligand-protein complex and the interaction studies of the most active compounds, **5b **and **5k** at the MAO-A protein binding pocket have been depicted in Figure 3(Fig. 3) and 4(Fig. 4). Docking studies inferred that Ala 68, Tyr69 and Phe352 are some important residues interacting at the MAO-A binding pocket. It was evident that at 2-pyrazoline nucleus, N1 *p*-chlorobenzenesulfonyl substitution proved to be very useful and all the compounds having this substitution showed better activity than the N1 unsubstituted pyrazolines (Tripathi et al., 2016[40]). The presence of sulfonyl group at N1 position of 2-pyrazoline nucleus evidenced H-bonding interaction between sulfonyl oxygen and Ala68 and Tyr69 residues at the MAO-A binding pocket. It was demonstrated in previous works that ligands having such type of hydrogen bond interactions are well placed in the aromatic cage of the MAO protein possessing C5 furyl/ substituted phenyl ring and N1-benzenesulfonyl ring of 2-pyrazoline nucleus. The naphthyl/4-chlorophenyl rings showed, imperative pi-pi stacking interactions with Phe352 backbone residue. It was also evident that most of the interactions of PDB co-crystal ligand 2Z5X were conserved in compounds **5b **and **5k**. Furthermore, the predicted binding affinity (Glide gscores) of the prepared analogs complemented well with the *in vivo *anti-anxiety and antidepressant activities data.

The synthesized compounds were also tested for their probable neurotoxicological effects, such as motor coordination (rotarod test) and locomotor activity (actophotometer test). The obtained results suggested that these derivatives were completely free from severe neurotoxicity (motor co-ordinations and locomotor disturbances) threats at the evaluated doses (Table 2(Tab. 2)). In Rotarod test, the mean fall-off time of most of the mice was greater than 180 seconds, and hence these compounds may not affect the motor coordination of the animals. Also, the mean counts in actophotometer test in pre-dose studies, and after 1 hour of dosing, were not significantly different and therefore, any probability of CNS stimulating or depressing effects of the tested compounds was ruled out. Additionally, most potent derivatives were evaluated for their acute toxicity at the corresponding doses. None of them showed any changes in behavioral pattern, convulsions, tremors, skin changes and death throughout the 14 days acute toxicity studies. Also, encouraging *in silico* pharmacokinetic properties (Table 3(Tab. 3)) were observed. Moreover, the screened compounds were not liable to cause carcinogenicity, mutagenicity, reproductive toxicity, acute toxicity and irritancy. Therefore, they were regarded to be safe as was corroborated by the computational programs such as LAZAR and OSIRIS (Table 4(Tab. 4)).

## Conclusion

This study concludes that the synthesized 2-pyrazoline analogs possess excellent to good antidepressant and anti-anxiety potential, as evaluated using various *in vivo *methods. The presence of C-3 naphthalen-1-yl substitution and C-5 furan-2-yl substitution at 2-pyrazoline nucleus was decisive for the antidepressant activity (**5k**). However, a polar substitution of 4-hydroxyphenyl group at 3^rd^ position (**5b**) was essential in eliciting anxiolytic activity. Nearly all the compounds were completely free from any neurotoxic indications, with a complementary pharmacokinetic behavior as predicted by *in silico *methods. Molecular docking experiments ascertained some imperative interactions of these compounds with the target protein, playing a pivotal role in neuropharmacology. Thus, in a nutshell, the synthesized 2-pyrazoline derivatives may prove their worth in the treatment and management of certain mental disorders, possibly through MAO-A inhibition. 

## Acknowledgements

The authors would like to thank Central Drugs Research Institute (CDRI), Lucknow, India and Department of Chemistry, Banasthali Vidyapith University, Banasthali, Rajasthan, India for providing the library and sophisticated analytical instrument facilities. We would extend our sincere gratitude to the All India Council for Technical Education (AICTE), New Delhi, India, for providing grant under the Research Promotion Scheme (Grant No.: 8023/RID/RPS/30 (Pvt.) 2011-12), through which the computational software facility has been made available at the host institute and the technical support team/application scientists of Schrödinger Inc. for their help during implementation of the project.

## Conflict of interest

No conflict of interest is reported by the authors.

## Figures and Tables

**Table 1:**
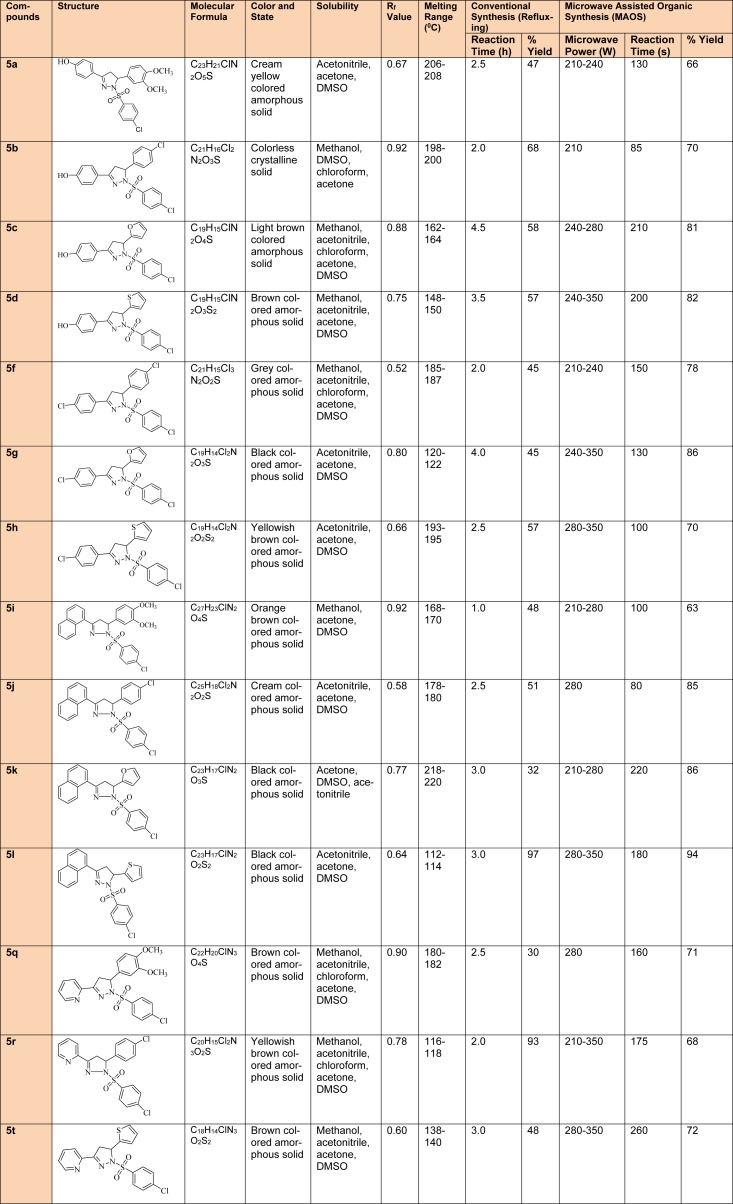
Comparative study of physicochemical properties of synthesized 1,3,5-trisubstituted-2-pyrazoline derivatives (5a-5t)

**Table 2:**
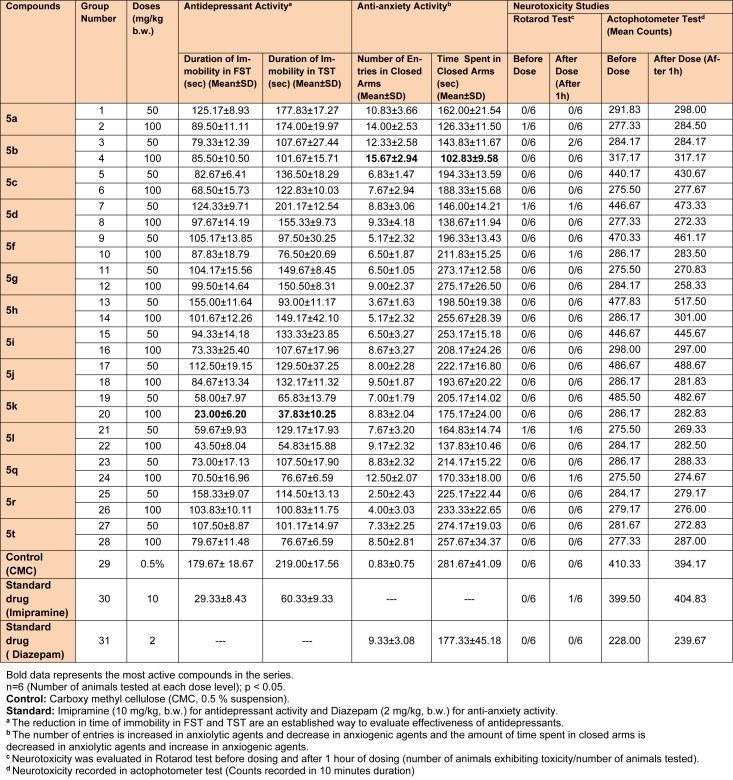
Data showing antidepressant, anti-anxiety and neurotoxicity studies of the synthesized 2-pyrazoline derivatives (5a-5t)

**Table 3:**
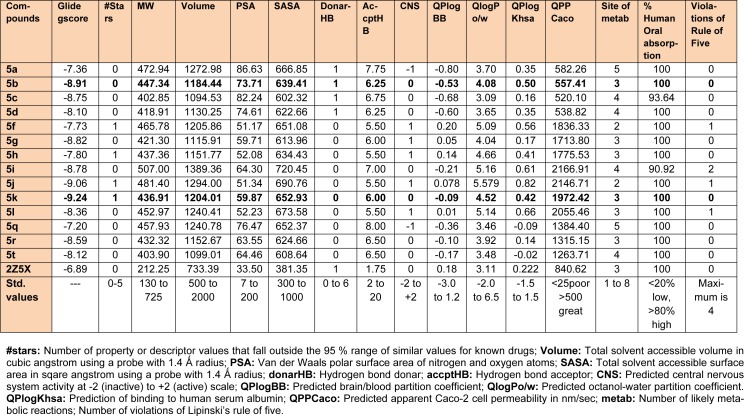
*In silico* prediction of binding affinity (Glide gscore) and ADME parameters of the synthesized 1,3,5-trisubstituted-2-pyrazoline derivatives (5a-5t)

**Table 4:**
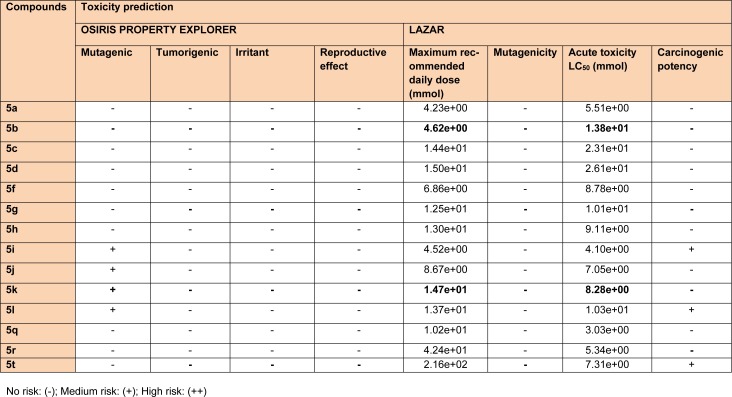
*In silico* toxicity prediction data of the synthesized 1,3,5-trisubstituted -2-pyrazoline derivatives (5a-5t)
